# Epidemiological and clinical burden of *Clostridioides difficile* infections and recurrences between 2015 − 2019: the RECUR Germany study

**DOI:** 10.1186/s12879-024-09218-y

**Published:** 2024-03-27

**Authors:** Aurore Tricotel, Ana Antunes, Adrian Wilk, Silvia Dombrowski, Hanna Rinta-Kokko, Fredrik L. Andersson, Subrata Ghosh

**Affiliations:** 1 IQVIA, Real World Solutions, Paris, France; 2 IQVIA, Global Database Studies, Real World Solutions, Lisbon, Portugal; 3 Team Gesundheit, Gesellschaft für Gesundheitsmanagement mbH, Essen, Germany; 4 IQVIA, Real World Solutions, Frankfurt, Germany; 5 IQVIA, Global Database Studies, Real World Solutions, Espoo, Finland; 6 Ferring Pharmaceuticals, Copenhagen, Denmark; 7 College of Medicine and Health, University College Cork, Cork, Ireland

**Keywords:** *Clostridioides difficile* infection, Recurrent *Clostridioides difficile* infection, Clinical, Epidemiological, Real-world study, Germany

## Abstract

**Background:**

This real-world study assessed the epidemiology and clinical complications of *Clostridioides difficile* infections (CDIs) and recurrences (rCDIs) in hospital and community settings in Germany from 2015 − 2019.

**Methods:**

An observational retrospective cohort study was conducted among adult patients diagnosed with CDI in hospital and community settings using statutory health insurance claims data from the BKK database. A cross-sectional approach was used to estimate the annual incidence rate of CDI and rCDI episodes per 100,000 insurants. Patients’ demographic and clinical characteristics were described at the time of first CDI episode. Kaplan-Meier method was used to estimate the time to rCDIs and time to complications (colonic perforation, colectomy, loop ileostomy, toxic megacolon, ulcerative colitis, peritonitis, and sepsis). A Cox model was used to assess the risk of developing complications, with the number of rCDIs as a time-dependent covariate.

**Results:**

A total of 15,402 CDI episodes were recorded among 11,884 patients. The overall incidence of CDI episodes declined by 38% from 2015 to 2019. Most patients (77%) were aged ≥ 65 years. Around 19% of CDI patients experienced at least one rCDI. The median time between index CDI episode to a rCDI was 20 days. The most frequent complication within 12-months of follow-up after the index CDI episode was sepsis (7.57%), followed by colectomy (3.20%). The rate of complications increased with the number of rCDIs. The risk of any complication increased by 31% with each subsequent rCDI (adjusted hazard ratio [HR]: 1.31, 95% confidence interval: 1.17;1.46).

**Conclusions:**

CDI remains a public health concern in Germany despite a decline in the incidence over recent years. A substantial proportion of CDI patients experience rCDIs, which increase the risk of severe clinical complications. The results highlight an increasing need of improved therapeutic management of CDI, particularly efforts to prevent rCDI.

**Supplementary Information:**

The online version contains supplementary material available at 10.1186/s12879-024-09218-y.

## Background

*Clostridioides difficile* is a leading cause of healthcare-associated infectious diarrhoea [1, 2], with 20−30% of *C. difficile* infections (CDIs) being acquired in community settings [3]. CDI symptoms range from mild diarrhoea to severe complications, including colitis, toxic megacolon, colonic perforation, and sepsis [4, 5]. Around a quarter of CDI patients experience a recurrence (rCDI), of which 40–65% experience multiple rCDIs [6]. Frequent rCDIs lead to repeated hospitalisations, reduced health-related quality of life [7–9], and risk of death [10]. Common risk factors to CDI and rCDI include antibiotic use, advanced age, and admission to healthcare facilities [11]. CDI is routinely treated with antibiotics, including vancomycin and fidaxomicin (metronidazole may also be used only if vancomycin and fidaxomicin are not available), while surgical intervention and non-antimicrobial management (via faecal microbiota transplant) may be required for severe or unresponsive patients with multiple rCDIs [12, 13].

The European Centre for Disease Prevention and Control (ECDC) surveillance report for 2016–2017 reported a crude incidence density of 3.48 cases per 10,000 patient-days in acute-care hospitals [14]. Recurrences were reported in 6.4% of cases and these were almost twice as likely to develop complications when compared with non-recurrent cases [14]. A systematic review and meta-analysis of studies and surveillance reports between 2005 and 2015 estimated a median overall incidence rate (IR) of 4.00 CDI cases per 10,000 patient-days [15]. Among European countries, one of the highest median overall incidences was reported in Germany (7.00 CDI cases per 10,000 patient-days) [15]. Recent studies on the burden of hospitalised CDI in Germany over the last decade have shown a decline in the incidence of CDI [16, 17]. It is however expected that the burden of CDI is likely underestimated as the surveillance system in Germany (as in other countries) is based on hospitalised cases only [17].

The present study aims to generate real-world evidence on the epidemiology and clinical complications of CDI and rCDI both in hospital and community settings in Germany using claims data between 2015 and 2019.

## Materials and methods

### Study design and data source

This observational retrospective study used claims data from BKK insurants (i.e., members of *Betriebskrankenkassen*, company-based sickness funds), part of the German Statutory Health Insurance (SHI) scheme database covering about 90% of the German population (i.e., 73 million people). The SHI database includes primary and specialty care data linked to hospital data and covers registrations, out- and in-patient care, drug prescriptions in community settings, and other services. The BKK database contains anonymised data of 5 million insurants and is representative of the entire SHI-insured population in terms of age, sex, prescriptions, and hospital diagnoses.

In this study, a cross-sectional approach spanning a five-year period (January 1, 2015 to December 31, 2019) was used to describe the annual incidence of CDI episodes and patient characteristics. A pre-index period of 12 months was used to characterise patients at baseline, namely on comorbidities, comedications and other potential risk factors. The year 2020 was not considered in the study period due to the potential impact of the COVID-19 pandemic on hospitalisations and ambulatory care as well as on the timely impact on data to be retrieved from BKK.

A longitudinal approach was performed to estimate the time from index CDI to rCDI, time from an rCDI to subsequent rCDI, and to assess the rate and risk of clinical complications at 12 months of follow-up. Patients were censored at the time of death, loss to follow-up, or at the end of the study period (December 31, 2019), whichever occurred first.

### Study population

Adult patients (≥ 18 years) diagnosed with CDI (International Classification of Diseases version 10 [ICD-10] code: A04.7) in a hospital or community setting between 2015 and 2019 and with a valid database record at least 12 months before the index date were included. Patients experiencing a CDI episode within 60 days prior to index episode were excluded from the study.

In community settings, diagnoses were only available on quarterly basis. To rule out potential false positives, patients with ≥ 1 gastrointestinal condition(s) (ICD-10 codes A09, K52, A48, K50, K57) during the same or previous quarter of CDI diagnoses and patients with no record of biological tests for identification of bacterial toxin A or B, nor prescriptions of CDI-related antibiotics within the quarter of the diagnosis were excluded. A sensitivity analysis was conducted without excluding these patients to estimate the upper range of CDI incidence.

### Definitions of CDI, rCDI episodes and new occurrence of CDI

The first CDI episode reported during the study period was designated as the index episode. Index episodes were classified according to the setting of treatment (hospital [hospitalised CDI] or community setting [community-treated CDI]) and setting of infection (healthcare-, community-associated CDI or unknown) (Fig. S1).

A CDI episode occurring within 60 days from the first CDI episode was considered a rCDI. The same criterion was applied to a subsequent CDI episode (i.e., second and third rCDI) occurring within 60 days of the previous rCDI. rCDI episodes in hospital settings were identified using diagnostic codes. In community settings, these were identified using two consecutive prescriptions of antibiotics indicated for CDI (i.e., non-topical metronidazole, or vancomycin, or fidaxomicin) where the second prescription was considered as the rCDI start date. In case of the same antibiotic, the two prescriptions should have been prescribed within a 30–60-day interval.

A CDI episode occurring beyond 60 days from the previous episode (index or rCDI) was considered a new CDI occurrence. Only the first occurrence of a new CDI was considered in the study. New CDI episodes were identified either by an ICD-10 diagnosis, or a second prescription of CDI-related antibiotics, if occurring beyond 60 days from the previous episode.

For hospitalised CDI, hospital entry dates were considered the start dates of episodes, unless CDI was reported as a secondary diagnosis and the hospital stay was > 14 days, in which the episode start date was approximated either by the median length of hospital stays with a reported CDI diagnosis or discharge date, whichever occurred first. For community-treated CDI episodes, prescriptions of CDI-related antibiotics were used to approximate the episode start date. Records of biological tests ordered for identification of bacterial toxin A or B were used if prescriptions were missing.

### Data analysis

Analyses were conducted using SAS® version 9.4 and graphs were generated using R software version 4.0.5. Absolute numbers and percentages were computed for categorical variables while descriptive statistics (mean, standard deviation [SD], median, 25th and 75th percentiles, and minimum and maximum) were displayed for quantitative variables. A significance level of 5% was considered (*P* < 0.05 for two-tailed test) for all statistical comparisons.

Annual incidence of CDI episodes was calculated per 100,000 inhabitants using the respective adult population of BKK insurants per year as the denominator. Charlson Comorbidity Index (CCI) comorbidities were identified using algorithms (Table S1) and the age-adjusted CCI score was categorized into 0–6, 7–10 or ≥ 11 points [18].

Longitudinal analyses were restricted to the subpopulation of patients diagnosed before December 31, 2018 without any CDI episode within 6 months prior to the index CDI episode. Time in days between index CDI episode and first rCDI, and from the previous rCDI to the subsequent episode was described using the Kaplan-Meier (KM) estimator (median and interquartile range), and survival curves with corresponding 95% confidence intervals (95% CIs). The rate of first complication was calculated at 3, 6 and 12-months of follow-up, overall (i.e., for ‘any complication’) and for each complication. The KM method was used to estimate the cumulative incidence of complications over follow-up. The risk of developing any complication at 12-month follow-up was estimated using Cox regression models where the number of rCDIs was treated as a time-dependent covariate, adjusting for potential confounders. Covariates that met the significance level of < 0.05 were added into the multivariate model using backward selection.

## Results

### Study participants

Following the screening of 12,330 patients with ≥ 1 CDI diagnosis recorded between January 1, 2015 and December 31, 2019, a total of 11,884 (96.4%) patients (Table S2) were included in the study. At the index CDI episode, most patients (88.3%; *n* = 10,491) were treated in hospital settings.

### Incidence and incidence rate of index CDI, rCDI, and new CDI episodes

A total of 15,402 CDI episodes (of any type) were observed in the cross-sectional cohort (Table 1). A decline of approximately 38% in the IR of CDI episodes (of any type) was observed between 2015 and 2019 (123.88 and 77.06 episodes per 100,000 insurants, respectively). A similar decrease was observed for index CDI (from 96.56 to 59.79 per 100,000 insurants) and first rCDI (from 17.8 to 10.61 per 100,000 insurants) episodes.

**Table 1:** Incidence and incidence rate of CDI episodes from 2015 to 2019

		2015	2016	2017	2018	2019	2015–2019
Population	*N*	2,909,959	2,978,605	3,059,526	3,093,212	3,137,848	15,179,150
CDI episodes (any type)	*n*	3,605	3,317	3,132	2,930	2,418	15,402
	IR	123.88	111.36	102.37	94.72	77.06	101.47
***Type of CDI episodes***							
• Index CDIs	*n*	2,810	2,536	2,414	2,248	1,876	11,884
	IR	96.56	85.14	78.9	72.68	59.79	78.29
• First rCDIs	*n*	518	448	426	397	333	2,122
	IR	17.8	15.04	13.92	12.83	10.61	13.98
• Second rCDIs	*n*	120	112	105	104	72	513
	IR	4.12	3.76	3.43	3.36	2.29	3.38
• Third or more rCDIs	*n*	50	53	49	40	28	220
	IR	1.72	1.78	1.6	1.29	0.89	1.45
• New occurrences of CDI	*n*	107	168	138	141	109	663
	IR	3.68	5.64	4.51	4.56	3.47	4.37
CDI episodes (any type, sensitivity analysis)^**a**^	*n*	4,546	4,202	3,952	3,733	3,142	19,575
	IR	156.22	141.07	129.17	120.68	100.13	128.96

^**a**^ Sensitivity analysis: Number of CDI episodes when potential false positive cases recorded in community setting are included in the analysis

CDI: *Clostridioides difficile* infection; IR: Incidence rate; N: Number of patients; n: Number of CDI episodes; rCDI: Recurrent *Clostridioides difficile* infection

When accounting for CDI patients in community settings that might be false positives (sensitivity analysis), the opposite was observed, with the total number of CDI episodes increasing from 15,402 to 19,575 over the study period. However, a similar IR decline of approximately 36% was observed from 156.22 to 100.13 CDI episodes per 100,000 insurants in 2015 and 2019, respectively.

Table 2 characterises patients according to the number of rCDIs (1, 2, ≥ 3) over the study period. Overall, 17.86% (*n* = 2,122) patients experienced ≥ 1 rCDI; 13.54% (*n* = 1,609) 1 rCDI, 3.03% (*n* = 360) 2 rCDIs, and 1.29% (*n* = 153) ≥ 3 rCDIs. Among those with ≥ 1 rCDI (*n* = 2,122 patients), 24.18% (*n* = 513) experienced a second rCDI, of whom 29.82% (*n* = 153) had at least a third rCDI (data not shown).

**Table 2:** Number and percentage of patients with rCDI episodes from 2015 to 2019

		2015–2019
Total patients	*N*	11,884
Patients with rCDIs (any number)	*n* (%)	2,122 (17.86%)
• Patients with 1 rCDI	*n* (%^**a**^)	1,609 (13.54%)
• Patients with 2 rCDIs	*n* (%^**a**^)	360 (3.03%)
• Patients with ≥ 3 rCDIs	*n* (%^**a**^)	153 (1.29%)
• Number of rCDIs per patient	Mean (SD)	0.24 (0.60)
	Min; Max	0; 8

^**a**^ Proportion estimated with respect to all CDI patients

rCDI: Recurrent *Clostridioides difficile* infection; Max: Maximum; Min: Minimum; N: Number of patients; SD: Standard deviation

### Demographic and clinical characteristics of CDI and rCDI patients

Table 3 presents patients’ demographic and clinical characteristics. The mean age of CDI patients was 73.23 years (median [Q1; Q3]: 78 [66.00; 84.00]). Most CDI patients were aged ≥ 65 years (76.79%; *n* = 9,126). This proportion increased to 81.86% (*n* = 1,737) among patients with ≥ 1 rCDI. A slightly higher proportion of women (52.29%) was observed, which increased to 57.52% in patients with ≥ 3 rCDIs. The median age-adjusted CCI registered at index date ranged from 8 to 9 across the different subgroups and was higher among patients with two or more rCDIs. Most patients (92.69%, *n* = 11,015) had pre-existing comorbidities, with chronic kidney disease and renal failure (49.35%, *n* = 5,865), heart failure (43.70%, *n* = 5,193), and ischaemic heart disease (37.62%, *n* = 4,471) being the most reported. The proportion of patients with comorbidities increased with the number of rCDIs. During the pre-index period, a high proportion of CDI patients were treated with antibiotics (48.76%; *n* = 5,795) and PPIs (58.74%; *n* = 6,981). A gastrointestinal procedure was recorded among 11.91% (*n* = 1,415) of patients.

**Table 3:** Demographic and clinical characteristics of CDI patients at index (overall and by number of rCDIs)

	All CDI patients	Patients with 1 rCDI^a^	Patients with 2 rCDIs^a^	Patients with ≥ 3 rCDIs^a^	Patients with ≥ 1 rCDIs	Non-rCDI patients (0 rCDI)^a^
**Total number of patients (** *N* **)**	11,884	1,609	360	153	2,122	9,762
**Age at index date (years)**						
Mean (SD)	73.23 (15.82)	74.49 (14.59)	76.81 (13.01)	77.08 (11.28)	75.07 (14.15)	72.84 (16.14)
Median (Q1; Q3)	78 (66.00; 84.00)	78 (68.00; 84.00)	80 (72.00; 85.00)	80 (73.00; 84.00)	79 (69.00; 85.00)	77 (65.00; 84.00)
**Age group at index, ** *n* ** (%)**						
18–64 years	2,758 (23.21%)	314 (19.52%)	48 (13.33%)	23 (15.03%)	385 (18.14%)	2,373 (24.31%)
≥ 65 years	9,126 (76.79%)	1,295 (80.48%)	312 (86.67%)	130 (84.97%)	1,737 (81.86%)	7,389 (75.69%)
**Gender, ** *n * **(%)**						
Women	6,214 (52.29%)	844 (52.45%)	201 (55.83%)	88 (57.52%)	1,133 (53.39%)	5,081 (52.05%)
Men	5,670 (47.71%)	765 (47.55%)	159 (44.17%)	65 (42.48%)	989 (46.61%)	4,681 (47.95%)
**Geographic region, ** *n * **(%)**						
North	1,519 (12.78%)	217 (13.49%)	55 (15.28%)	19 (12.42%)	291 (13.71%)	1,228 (12.58%)
West	3,767 (31.70%)	509 (31.63%)	109 (30.28%)	65 (42.48%)	683 (32.19%)	3,084 (31.59%)
East	3,029 (25.49%)	419 (26.04%)	90 (25.00%)	35 (22.88%)	544 (25.64%)	2,485 (25.46%)
South	3,535 (29.75%)	459 (28.53%)	104 (28.89%)	33 (21.57%)	596 (28.09%)	2,939 (30.11%)
Unknown	34 (0.29%)	5 (0.31%)	2 (0.56%)	1 (0.65%)	8 (0.38%)	26 (0.27%)
**Age-adjusted Charlson Comorbidity Index**						
Mean (SD)	8.00 (3.96)	8.03 (3.83)	8.77 (3.43)	8.69 (3.09)	8.21 (3.73)	7.95 (4.01)
Median (Q1; Q3)	8 (5.00; 11.00)	8 (6.00; 11.00)	9 (7.00; 11.00)	9 (7.00; 10.00)	8 (6.00; 11.00)	8 (5.00; 11.00)
**Age-adjusted Charlson Comorbidity Index categories, ** *n * **(%)**						
Score 0–6	3,931 (33.08%)	514 (31.95%)	85 (23.61%)	37 (24.18%)	636 (29.97%)	3,295 (33.75%)
Score 7–10	4,762 (40.07%)	688 (42.76%)	158 (43.89%)	79 (51.63%)	925 (43.59%)	3,837 (39.31%)
Score ≥ 11	3,191 (26.85%)	407 (25.30%)	117 (32.50%)	37 (24.18%)	561 (26.44%)	2,630 (26.94%)
**Pre-index comorbid conditions, ** *n * **(%)**						
Any condition below	11,015 (92.69%)	1,484 (92.23%)	346 (96.11%)	147 (96.08%)	1,977 (93.17%)	9,038 (92.58%)
Crohn’s disease	182 (1.53%)	15 (0.93%)	0 (0.00%)	0 (0.00%)	15 (0.71%)	167 (1.71%)
Ulcerative colitis	310 (2.61%)	40 (2.49%)	8 (2.22%)	4 (2.61%)	52 (2.45%)	258 (2.64%)
Other noninfective gastroenteritis and colitis	1,741 (14.65%)	233 (14.48%)	38 (10.56%)	17 (11.11%)	288 (13.57%)	1,453 (14.88%)
Toxic megacolon	11 (0.09%)	0 (0.00%)	1 (0.28%)	0 (0.00%)	1 (0.05%)	10 (0.10%)
Colonic perforation	87 (0.73%)	15 (0.93%)	3 (0.83%)	0 (0.00%)	18 (0.85%)	69 (0.71%)
Peritonitis	277 (2.33%)	31 (1.93%)	3 (0.83%)	3 (1.96%)	37 (1.74%)	240 (2.46%)
Diabetes mellitus type 1	566 (4.76%)	86 (5.34%)	12 (3.33%)	6 (3.92%)	104 (4.90%)	462 (4.73%)
Malignant neoplasms	3,500 (29.45%)	445 (27.66%)	102 (28.33%)	43 (28.10%)	590 (27.80%)	2,910 (29.81%)
Haematological malignancy	550 (4.63%)	61 (3.79%)	10 (2.78%)	7 (4.58%)	78 (3.68%)	472 (4.84%)
Immunosuppression	4,442 (37.38%)	573 (35.61%)	122 (33.89%)	52 (33.99%)	747 (35.20%)	3,695 (37.85%)
Chronic kidney disease and renal failure	5,865 (49.35%)	823 (51.15%)	200 (55.56%)	85 (55.56%)	1,108 (52.21%)	4,757 (48.73%)
Chronic lower respiratory diseases	3,967 (33.38%)	538 (33.44%)	122 (33.89%)	51 (33.33%)	711 (33.51%)	3,256 (33.35%)
Respiratory failure	2,646 (22.27%)	368 (22.87%)	62 (17.22%)	33 (21.57%)	463 (21.82%)	2,183 (22.36%)
Ischaemic heart disease	4,471 (37.62%)	594 (36.92%)	156 (43.33%)	62 (40.52%)	812 (38.27%)	3,659 (37.48%)
Heart failure	5,193 (43.70%)	726 (45.12%)	175 (48.61%)	76 (49.67%)	977 (46.04%)	4,216 (43.19%)
Liver disease	2,520 (21.20%)	325 (20.20%)	67 (18.61%)	33 (21.57%)	425 (20.03%)	2,095 (21.46%)
Cerebrovascular disease	4,205 (35.38%)	577 (35.86%)	154 (42.78%)	58 (37.91%)	789 (37.18%)	3,416 (34.99%)
Dementia	3,121 (26.26%)	478 (29.71%)	124 (34.44%)	46 (30.07%)	648 (30.54%)	2,473 (25.33%)
**Pre-index treatments, medical procedures and consultations, ** *n * **(%)**						
*Pre-index medication* ^**b**^						
Antibiotics	5,795 (48.76%)	753 (46.80%)	176 (48.89%)	65 (42.48%)	994 (46.84%)	4,801 (49.18%)
Laxatives	1,370 (11.53%)	180 (11.19%)	42 (11.67%)	18 (11.76%)	240 (11.31%)	1,130 (11.58%)
PPIs	6,981 (58.74%)	968 (60.16%)	228 (63.33%)	97 (63.40%)	1,293 (60.93%)	5,688 (58.27%)
H2-receptor antagonists	268 (2.26%)	34 (2.11%)	10 (2.78%)	1 (0.65%)	45 (2.12%)	223 (2.28%)
Selective immunosuppressants	178 (1.50%)	26 (1.62%)	3 (0.83%)	0 (0.00%)	29 (1.37%)	149 (1.53%)
TNF-α inhibitors	51 (0.43%)	5 (0.31%)	0 (0.00%)	0 (0.00%)	5 (0.24%)	46 (0.47%)
Interleukin inhibitors	12 (0.10%)	0 (0.00%)	1 (0.28%)	0 (0.00%)	1 (0.05%)	11 (0.11%)
Calcineurin inhibitors	137 (1.15%)	20 (1.24%)	2 (0.56%)	1 (0.65%)	23 (1.08%)	114 (1.17%)
Other immunosuppressants	224 (1.88%)	32 (1.99%)	4 (1.11%)	2 (1.31%)	38 (1.79%)	186 (1.91%)
Chemotherapies/ antineoplastic agents	709 (5.97%)	78 (4.85%)	21 (5.83%)	11 (7.19%)	110 (5.18%)	599 (6.14%)
*Pre-index medical procedures (gastrointestinal)* ^**c**^	1,415 (11.91%)	175 (10.88%)	43 (11.94%)	21 (13.73%)	239 (11.26%)	1,176 (12.05%)
*Pre-index hospitalisations*	9,114 (76.69%)	1,247 (77.50%)	291 (80.83%)	135 (88.24%)	1,673 (78.84%)	7,441 (76.22%)
*Pre-index primary care consultations*	11,806 (99.34%)	1,598 (99.32%)	359 (99.72%)	153 (100.00%)	2,110 (99.43%)	9,696 (99.32%)
*Pre-index outpatient consultations*	1,880 (15.82%)	243 (15.10%)	56 (15.56%)	30 (19.61%)	329 (15.50%)	1,551 (15.89%)
*Pre-index diagnosis tests*	1,387 (11.67%)	197 (12.24%)	41 (11.39%)	15 (9.80%)	253 (11.92%)	1,134 (11.62%)
*Pre-index admittances to ICU*	993 (8.36%)	138 (8.58%)	24 (6.67%)	13 (8.50%)	175 (8.25%)	818 (8.38%)
**Setting of treatment at index, ** *n * **(%)**						
Hospitalised	10,491 (88.28%)	1,396 (86.76%)	306 (85.00%)	127 (83.01%)	1,829 (86.19%)	8,662 (88.73%)
Community-treated	1,393 (11.72%)	213 (13.24%)	54 (15.00%)	26 (16.99%)	293 (13.81%)	1,100 (11.27%)
**Setting of infection at index, ** *n * **(%)**						
Healthcare-associated	2,174 (18.29%)	386 (23.99%)	103 (28.61%)	43 (28.10%)	532 (25.07%)	1,642 (16.82%)
Community-associated	4,519 (38.03%)	571 (35.49%)	111 (30.83%)	47 (30.72%)	729 (34.35%)	3,790 (38.82%)
Unknown	5,191 (43.68%)	652 (40.52%)	146 (40.56%)	63 (41.18%)	861 (40.57%)	4,330 (44.36%)

^**a**^ Non-rCDI patients (0 rCDI), Patients with 1 rCDI, Patients with 2 rCDIs, Patients with ≥ 3 rCDIs are subgroups of ‘all CDI patients’

^**b**^ including antibiotics (cephalosporins, fluoroquinolones, macrolides, penicillins, rifaximin, clindamycin), laxatives, PPIs, H2-receptor antagonists, selective immunosuppressants, TNF-α inhibitors, interleukin inhibitors, calcineurin inhibitors, other immunosuppressants, chemotherapies/ antineoplastic agents

^**c**^ including gastrointestinal surgeries (appendectomy, bariatric surgeries, bowel resection surgeries, subtotal colectomy, ileostomy) and nasogastric tube placement

CDI: *C. difficile* infection; ICU: Intensive care unit; PPIs: Proton-pump inhibitors; Q1: 25th percentile; Q3: 75th percentile; rCDI: Recurrent *C. difficile* infection, SD: Standard deviation; TNF-α: Tumour necrosis factor-alpha

Of the 56.32% (*n* = 6,693) of patients with a known setting of infection, 67.52% (*n* = 4,519) were classified as having a community-associated index CDI episode and 32.48% (*n* = 2,174) as having a healthcare-associated index CDI episode. The setting of infection was unknown in 43.68% of patients (*n* = 5,191).

### Time between CDI and rCDI episodes

Table 4 shows the time from the index CDI episode to the first rCDI, and the time from the previous rCDI to the next rCDI. In the subset of patients for longitudinal analysis (*n* = 9,977), 18.06% (*n* = 1,802) experienced ≥ 1 rCDI event after a median time of 20 days from the index CDI episode. The time to the subsequent recurrent event was consistent until the fourth episode of rCDI, ranging between a median of 20 and 22 days (Table 4, Fig. S2).

**Table 4:** Time from index CDI episode to first rCDI, and from previous rCDI to next rCDI

	Indicator	Measure
Total number of CDI patients	*N*	9,977
Patients with ≥ 1 rCDI	*n* (%)	1,802 (18.06%)
Time from index CDI to first rCDI (days)	Median time in days to event [95% CI]	20 [19.00; 21.00]
	Q1; Q3	10.00; 33.00
Patients with ≥ 2 rCDIs	*n* (%)	446 (4.47%)
Time from first rCDI to second rCDI (days)	Median time to event [95% CI]	22 [21.00; 25.00]
	Q1; Q3	12.00; 35.00
Patients with ≥ 3 rCDIs	*n* (%)	134 (1.34%)
Time from second rCDI to third rCDI (days)	Median time to event [95% CI]	21 [17.00; 24.00]
	Q1; Q3	9.00; 35.00
Patients with at ≥ 4rCDIs	*n* (%)	39 (0.39%)
Time from third rCDI to fourth rCDI (days)	Median time to event [95% CI]	22 [18.00; 28.00]
	Q1; Q3	13.00; 32.00
	Min; Max	4; 59

*Data presented until fourth rCDI.

CDI: *C. difficile* infection; CI: Confidence interval; Max: Maximum; Min: Minimum; Q1: 25th percentile; Q3: 75th percentile; rCDI: Recurrent *C. difficile* infection

### Rate of complications among CDI and rCDI patients

Table 5 presents the rates of complications among the subpopulation of patients for longitudinal analysis (*n* = 9,977) at 3-, 6- and 12-months of follow-up. The most common complication at 12-months of follow-up was sepsis (7.57%, *n* = 755), followed by colectomy (3.20%, *n* = 319), and ulcerative colitis (1.13%, *n* = 113). All other complications were observed in < 1% of patients. The rate of complications increased over the follow-up time. Among CDI patients, 8.40% (*n* = 838) had a complication (any type) at 3-months of follow-up, which increased to 10.34% (*n* = 1,032) and 12.43% (*n* = 1,240) at 6- and 12-months of follow-up, respectively. The rate of complications was higher among patients with rCDIs and increased at each subsequent episode (any complication at 12-months of follow-up observed in 14.75%, 13.78%, and 23.13% of patients with 1, 2 and ≥ 3 rCDIs, respectively).

**Table 5:** Rate of first complication at 3-, 6- and 12-months, overall and stratified by rCDIs

	Months (M) of follow-up	CDI patients	Patients without rCDI (0 rCDI)	Patients with 1 rCDI	Patients with 2 rCDI	Patients with ≥ 3 rCDI	Patients with ≥ 1 rCDI
**Number of patients**		9,977	8,175	1,356	312	134	1,802
**Any complication**	3 M	838 (8.40%)	636 (7.78%)	148 (10.91%)	34 (10.90%)	20 (14.93%)	202 (11.21%)
	6 M	1,032 (10.34%)	791 (9.68%)	174 (12.83%)	39 (12.50%)	28 (20.90%)	241 (13.37%)
	12 M	1,240 (12.43%)	966 (11.82%)	200 (14.75%)	43 (13.78%)	31 (23.13%)	274 (15.21%)
**Colonic perforation**	3 M	9 (0.09%)	6 (0.07%)	3 (0.22%)	0 (0.00%)	0 (0.00%)	3 (0.17%)
	6 M	15 (0.15%)	11 (0.13%)	4 (0.29%)	0 (0.00%)	0 (0.00%)	4 (0.22%)
	12 M	19 (0.19%)	14 (0.17%)	5 (0.37%)	0 (0.00%)	0 (0.00%)	5 (0.28%)
**Colectomy**	3 M	197 (1.97%)	151 (1.85%)	36 (2.65%)	7 (2.24%)	3 (2.24%)	46 (2.55%)
	6 M	247 (2.48%)	192 (2.35%)	43 (3.17%)	7 (2.24%)	5 (3.73%)	55 (3.05%)
	12 M	319 (3.20%)	258 (3.16%)	47 (3.47%)	8 (2.56%)	6 (4.48%)	61 (3.39%)
**Loop ileostomy**	3 M	21 (0.21%)	18 (0.22%)	3 (0.22%)	0 (0.00%)	0 (0.00%)	3 (0.17%)
	6 M	31 (0.31%)	26 (0.32%)	4 (0.29%)	0 (0.00%)	1 (0.75%)	5 (0.28%)
	12 M	40 (0.40%)	33 (0.40%)	6 (0.44%)	0 (0.00%)	1 (0.75%)	7 (0.39%)
**Toxic megacolon**	3 M	15 (0.15%)	10 (0.12%)	4 (0.29%)	1 (0.32%)	0 (0.00%)	5 (0.28%)
	6 M	15 (0.15%)	10 (0.12%)	4 (0.29%)	1 (0.32%)	0 (0.00%)	5 (0.28%)
	12 M	17 (0.17%)	12 (0.15%)	4 (0.29%)	1 (0.32%)	0 (0.00%)	5 (0.28%)
**Ulcerative colitis**	3 M	83 (0.83%)	58 (0.71%)	21 (1.55%)	2 (0.64%)	2 (1.49%)	25 (1.39%)
	6 M	95 (0.95%)	68 (0.83%)	22 (1.62%)	2 (0.64%)	3 (2.24%)	27 (1.50%)
	12 M	113 (1.13%)	84 (1.03%)	24 (1.77%)	2 (0.64%)	3 (2.24%)	29 (1.61%)
**Peritonitis**	3 M	63 (0.63%)	52 (0.64%)	7 (0.52%)	1 (0.32%)	3 (2.24%)	11 (0.61%)
	6 M	81 (0.81%)	66 (0.81%)	10 (0.74%)	1 (0.32%)	4 (2.99%)	15 (0.83%)
	12 M	93 (0.93%)	76 (0.93%)	12 (0.88%)	1 (0.32%)	4 (2.99%)	17 (0.94%)
**Sepsis**	3 M	497 (4.98%)	373 (4.56%)	86 (6.34%)	23 (7.37%)	15 (11.19%)	124 (6.88%)
	6 M	621 (6.22%)	471 (5.76%)	102 (7.52%)	29 (9.29%)	19 (14.18%)	150 (8.32%)
	12 M	755 (7.57%)	577 (7.06%)	123 (9.07%)	32 (10.26%)	23 (17.16%)	178 (9.88%)

CDI: *C. difficile* infection; rCDI: Recurrent *C. difficile* infection

The risk of developing any complication increased by 1.31 times with each subsequent rCDI (adjusted hazard ratio [HR]: 1.31, 95% CI: 1.17; 1.46) (Table 6). Patients treated in hospital settings had two times higher risk of developing complications in comparison to those treated in community settings (adjusted HR: 2.18, 95% CI: 1.72; 2.77). Males, patients with pre-index comorbidities, with pre-index medications, gastrointestinal procedures, hospitalisations, and outpatient consultations, and intensive care unit (ICU) admissions were also at higher risk of complications.

**Table 6:** Risk of any complication at 12-months of follow-up (time-dependent analysis)

Variable	CDI patients		Unadjusted model			Adjusted model^c^	
	*N*	HR	95% CI for HR	*P*-value	HR	95% CI for HR	*P*-value
**rCDI**							
x per number increase	9,977	1.31	(1.17; 1.46)	< 0.001	1.31	(1.17; 1.46)	**< 0.001**
**Gender**							
Female	5,217	-	-	-	1.00		
Male	4,760	-	-	-	1.26	(1.12; 1.41)	**< 0.001**
**Baseline characteristics**							
Pre-index medications^**a**^	7,990	-	-	-	1.42	(1.21; 1.67)	**< 0.001**
Pre-index medical procedures^**b**^	1,168	-	-	-	1.59	(1.37; 1.84)	**< 0.001**
Pre-index hospitalisations	7,665	-	-	-	1.36	(1.16; 1.59)	**< 0.001**
Pre-index primary care consultations	9,917	-	-	-	0.54	(0.29; 0.99)	**0.048**
Pre-index outpatient consultations	1,557	-	-	-	1.25	(1.08; 1.43)	**0.002**
Pre-index admittances to ICU	825	-	-	-	1.25	(1.05; 1.5)	**0.013**
Pre-index comorbidities (at least one, any of them)	9,253	-	-	-	1.8	(1.33; 2.44)	**< 0.001**
**Setting of treatment**							
Community-treated	1,161	-	-	-	1.00		
Hospitalised	8,816	-	-		2.18	(1.72; 2.77)	**< 0.001**

^**a**^ including antibiotics (cephalosporins, fluoroquinolones, macrolides, penicillins, rifaximin, clindamycin), laxatives, PPIs, H2-receptor antagonists, selective immunosuppressants, TNF-α inhibitors, interleukin inhibitors, calcineurin inhibitors, other immunosuppressants, chemotherapies/ antineoplastic agents

^**b**^ including gastrointestinal surgeries (appendectomy, bariatric surgeries, bowel resection surgeries, subtotal colectomy, ileostomy) and nasogastric tube placement

^**c**^ Variables included in the model: rCDIs, age, gender, pre-index medications, pre-index medical procedures, pre-index hospitalisations, pre-index primary care consultations, pre-index outpatient consultations, pre-index admittances to ICU, pre-index diagnosis tests, pre-index comorbidities (any), setting of treatment, setting of infection

CDI: *C. difficile* infection; CI: Confidence interval; HR: Hazard ratio; ICU: Intensive care unit; PPIs: proton-pump inhibitors; rCDI: Recurrent *C. difficile* infection; TNF-α: Tumour necrosis factor-alpha

## Discussion

A total of 15,402 CDI episodes were captured in 11,884 patients between 2015 and 2019. A decline in the annual IR of CDI and rCDI of approximately 38% was observed during the study period from 124 to 77 episodes per 100,000 insurants. Nearly a fifth of patients experienced a rCDI and the proportion of patients with a subsequent episode increased at each episode. Among patients with at least 1 rCDI, 24% experienced a second rCDI, of whom 30% experienced ≥ 3 rCDIs. Around 12% of CDI patients developed clinical complications within 12 months of follow-up, with sepsis being the most frequently observed. Complications were more common in patients with multiple rCDIs, being observed in 23% of patients with ≥ 3 rCDIs. The risk of any complication at 12-month of follow-up increased by 31% with each rCDI. To ensure specificity of CDI episodes and avoid false positives, patients with a diagnosis recorded in community settings with concurrent gastrointestinal conditions that could have been treated with antibiotics with a CDI indication were excluded from the main analysis, along with patients with no record of relevant antibiotic prescriptions or biological tests. When these patients were included in the sensitivity analysis, an additional 4,173 (27%) CDI episodes were added.

Overall, the results are aligned with recent data on the burden of hospitalised CDI from publicly available sources in Germany between 2010 and 2019, which has shown a steady decline from 2015 onwards, with 81 cases per 100,000 population observed in 2019 [17]. A study on hospital coding practices for CDI in Germany showed a decline of 52% in the number of cases recorded as a primary diagnosis (i.e., CDI as the reason for admission) and 49% of cases recorded as a secondary diagnosis (i.e., occurrence during the hospital stay or pre-existing condition), from 2015 to 2019 [16]. Several factors are likely to have contributed to the downward trend of CDI incidence in Germany over the study period, including hospital hygiene campaigns, implementation of antibiotic stewardship programs, among others.

Similar trends have been reported in other countries. In the United States (US), a decline from 149 to 121 cases per 100,000 persons was reported between 2015 and 2019 [19–24]. This has been attributed to a decrease in healthcare-associated CDI cases (as community-associated CDI remained stable) and explained by more judicious antibiotic use, especially fluoroquinolones [19–25]. Similar trends were also observed in France and Canada [26, 27]. However, a recent systematic review on the epidemiology of CDI and rCDI in 11 countries revealed no clear trends for incidence with time, with variations across countries [11]. Furthermore, the ECDC has emphasised the lack of a standardised CDI surveillance, which limits the identification of epidemiological changes in the region and consequently hinders CDI prevention and control [28].

In line with prior studies [2, 11, 29–32], CDI and rCDI were more frequently observed among older patients and in those with pre-existing comorbidities, such as chronic kidney disease, among others. Several factors contribute to the increased susceptibility to CDI and rCDI among the elderly, including immunosenescence, age-related alterations in the gut microbiome, overall poor health, increased hospitalisations/nursing home stays, and frequent use of antibiotics and PPIs [33]. Several studies established the role of antibiotics, PPIs, and gastrointestinal procedures as risk factors for CDI [3, 34, 35]. In the current study, a high proportion of CDI patients were prescribed antibiotics (49%) and PPIs (59%) during the pre-index period, and around 12% patients underwent a gastrointestinal procedure. However, the use of antibiotics is likely underestimated since hospital-dispensed treatments are not recorded in the BKK database. Like other studies, there was a higher proportion of female patients in our study [5, 36–38]. So far, no association has however been found between gender and susceptibility to CDI.

Almost 90% of CDI patients were treated in hospital settings, likely reflecting the management of the disease in Germany as well as patient characteristics such as older age, health status, or severity of disease. The substantially low proportion of patients treated in community settings could be partially explained by the exclusion of patients with concomitant gastrointestinal conditions, but misdiagnosis and misclassification can’t be ruled out either. Aligned with a previous study in the country, around 18% of patients experienced a recurrence a median 20 days after the index CDI episode. In that study, the risk of developing a second and third episode increased to 28.4% and 30.2%, respectively [30]. A 25% probability of developing a first rCDI after an initial infection has also been reported [37]. A more recent study found lower rates of rCDI rates probably due to the use of sub-codes to specify recurrences, which were not applied in this study.

Sepsis and colectomy were the most frequently recorded clinical complications with complications being higher in those with recurrences. Sepsis occurred in 7.6% of CDI patients vs. 17.16% in patients with ≥ 3 rCDIs. A real-world study in the US reported a similar pattern but with even higher rates of complications. Sepsis was observed in 16.5% of CDI patients without rCDIs at 12-months of follow-up, increasing to 27.3%, 33.1%, and 43.3% in patients with 1, 2, and 3 + rCDIs, respectively. Likewise, subtotal colectomy or diverting loop ileostomy occurred in 4.6% of patients with a first CDI episode, increasing with the number of recurrences to 7.3%. 8.9%, and 10.5% of patients with 1, 2, and 3 + rCDIs, respectively [36].

When estimating the risk of any complication at 12-months, the current study showed an increased risk of 31% with each subsequent rCDI. Complications were twice as likely to occur among patients treated in hospitals during the index episode compared to those treated in community settings, suggesting a more severe and frail patient population. Although the outcome of interest included any complication, the results were likely driven by sepsis, the most frequently reported complication. However, it should be noted that whether there is a causative relationship between CDI or rCDI and the incidence of complications remains unclear. Furthermore, complications may not necessarily develop after CDI diagnosis but could also be the relapse or exacerbation of previous complications.

The use of claims data from the German BKK database proved to be useful in capturing CDI and rCDI cases, and to provide further insights on the disease at the community level, which is not captured in the traditional hospital-based surveillance system. However, despite the development of an algorithm to ascertain the setting of infection, 43% of CDI episodes were classified as unknown, which limited the interpretation of the results. Like other studies relying on existing data, the primary intention of collecting claims data is not research, and some data may not be recorded or may be misclassified or miscoded, leading to bias. Further, diagnostic data are available in the BKK database only on a quarterly basis. Thus, patients with other gastrointestinal conditions and those who were not prescribed any antibiotic indicated for CDI, nor a test for identification of bacterial toxin A or B were excluded. This methodological choice probably led to an underestimation of the cases in the country.

## Conclusions

This real-world study provided an overview on the epidemiology and clinical complications of CDI and rCDI in Germany. Frequent rCDIs represent a particular concern, being associated with an increased risk of clinical complications. The findings demonstrate not only the urgent need to break the cycle of CDI and rCDI through the development of effective and innovative therapies but also to improve current surveillance systems to include community-treated cases.

### Electronic supplementary material

Below is the link to the electronic supplementary material.

Supplementary Material 1

## Data Availability

The data that support the findings of this study are available from statutory sickness funds but restrictions apply to the availability of these data, which were used under license for the current study, and are not publicly available. Data are available from the authors upon reasonable request, and with permission of data-providing sickness funds.

## Abbreviations

BKK: *Betriebskrankenkassen*
CCI: Charlson Comorbidity Index
CDI: *Clostridioides difficile* infection
CI: Confidence interval
ECDC: European Centre for Disease Prevention and Control
ICD-10: International Classification of Diseases version 10
IR: Incidence rate
KM: Kaplan-Meier
rCDI: Recurrent *Clostridioides difficile* infection
SD: Standard deviation
SHI: Statutory Health Insurance
HR: Hazard ratio
ICU: Intensive care unit
PPI: Proton-pump inhibitor
US: United States
