# Supplementary material for: Epidemiological and clinical burden of Clostridioides difficile infections and recurrences between 2015 − 2019: the RECUR Germany study
Source: BMC Infect Dis. 2024 Mar 27;24:357. doi: 10.1186/s12879-024-09218-y (PMC10976771; doi:10.1186/s12879-024-09218-y)
Supplement: Supplementary file 1 — Supplementary Material 1 [file 12879_2024_9218_MOESM1_ESM.docx]

**Supplementary Data**

**Tables**

**Table S1** List of ICD-10 codes for CDI, relevant complications, pre-index comorbidities and Charlson Comorbidity Index (CCI) comorbidities

| **ICD-10 codes list** | | |
| --- | --- | --- |
| **Conditions** | **ICD-10 code** | **ICD-10-GM code** |
| **CDI** | | |
| Enterocolitis due to *Clostridioides difficile* | A04.7 |  |
| **Complications/Gastrointestinal conditions** | | |
| Crohn’s disease | K50 |  |
| Ulcerative colitis | K51 |  |
| Other noninfective gastroenteritis and colitis | K52 |  |
| Colonic perforation (perforation of intestine– nontraumatic) | K63.1 |  |
| Toxic megacolon |  | K59.3 |
| Colectomy |  | Z90.4 |
| Ileostomy | Z43.2 |  |
| Peritonitis | K65 |  |
| Sepsis | A40, A41 |  |
| **Other conditions** | | |
| Chronic kidney disease and renal failure | N17-N19 |  |
| Diabetes mellitus type I | E10 |  |
| Liver disease | K70-K77 |  |
| Chronic lower respiratory diseases | J40-J47 |  |
| Respiratory failure | J96 |  |
| Ischaemic heart disease | I20-I25 |  |
| Heart failure | I11.0, I13.0, I13.2, I50 |  |
| Cerebrovascular disease | G45-G46, I60-I69 |  |
| Dementia | F00-F03, F05.1, G30, G31.1 |  |
| **Immunosuppression^a^** | | |
| Chemotherapy for solid tumours or malignant hemopathies | Z51.1, Z51.2, Z08.2, Z54.2 |  |
| Haematological malignancies | C81-C96 |  |
| Solid neoplasms | C00-C75 |  |
| Antineoplastic and immunosuppressive drugs | T45.1 | *ATC codes L01 and L04 were used.* |
| Kidney transplant recipient | Z94.0 |  |
| Heart transplant recipient | Z94.1 |  |
| Lung transplant recipient | Z94.2 |  |
| Heart and lungs transplants recipient | Z94.3 |  |
| Liver transplant recipient | Z94.4 |  |
| Pancreas transplant recipient |  | Z94.88 |
| Intestinal transplant recipient |  | Z94.88 |
| Allogenic stem-cell transplant recipient |  | Z94.80, Z94.81 |
| Graft rejection | T86 |  |
| HIV infection and AIDS | B20-B24 |  |
| Immune deficiency | D80-D84 |  |
| Agranulocytosis | D70 |  |
| Bone-marrow aplasia and others | D60-D61 |  |
| Antineoplastic chemotherapy | Z51.1, Z51.2, Z92.6 |  |
| Antineoplastic radiation therapy | Z51.0, Z54.1 |  |
| **Additional ICD-10 codes used for exclusion criteria** | | |
| Other gastroenteritis and colitis of infectious and unspecified origin | A09 |  |
| Other bacterial diseases, not elsewhere classified | A48 |  |
| Diverticular disease of intestine | K57 |  |
| **Charlson Comorbidity Index (CCI) comorbidities^b^** | | |
| **Comorbidities** | **ICD-10 code** | |
| Myocardial infarction | I21.x, I22.x, I25.2 | |
| Congestive heart failure | I09.9, I11.0, I13.0, I13.2, I25.5, I42.0, I42.5–I42.9, I43.x, I50.x, P29.0 | |
| Peripheral vascular disease | I70.x, I71.x, I73.1, I73.8, I73.9, I77.1, I79.0, I79.2, K55.1, K55.8, K55.9, Z95.8, Z95.9 | |
| Cerebrovascular disease | G45.x, G46.x, H34.0, I60.x–I69.x | |
| Dementia | F00.x–F03.x, F05.1, G30.x, G31.1 | |
| Chronic pulmonary disease | I27.8, I27.9, J40.x–J47.x, J60.x–J67.x, J68.4, J70.1, J70.3 | |
| Rheumatic disease | M05.x, M06.x, M31.5, M32.x–M34.x, M35.1, M35.3, M36.0 | |
| Peptic ulcer disease | K25.x–K28.x | |
| Mild liver disease | B18.x, K70.0–K70.3, K70.9, K71.3–K71.5, K71.7, K73.x, K74.x, K76.0, K76.2–K76.4, K76.8, K76.9, Z94.4 | |
| Diabetes without chronic complication | E10.0, E10.1, E10.6, E10.8, E10.9, E11.0, E11.1, E11.6, E11.8, E11.9, E12.0, E12.1, E12.6, E12.8, E12.9, E13.0, E13.1, E13.6, E13.8, E13.9, E14.0, E14.1, E14.6, E14.8, E14.9 | |
| Diabetes with chronic complication | E10.2–E10.5, E10.7, E11.2–E11.5, E11.7, E12.2–E12.5, E12.7, E13.2−E13.5, E13.7, E14.2–E14.5, E14.7 | |
| Hemiplegia or paraplegia | G04.1, G11.4, G80.1, G80.2, G81.x, G82.x, G83.0–G83.4, G83.9 | |
| Renal disease | I12.0, I13.1, N03.2–N03.7, N05.2–N05.7, N18.x, N19.x, N25.0, Z49.0−Z49.2, Z94.0, Z99.2 | |
| Any malignancy, including lymphoma and leukaemia, except malignant neoplasm of skin | C00.x–C26.x, C30.x–C34.x, C37.x–C41.x, C43.x, C45.x–C58.x, C60.x–C76.x, C81.x–C85.x, C88.x, C90.x–C97.x | |
| Moderate or severe liver disease | I85.0, I85.9, I86.4, I98.2, K70.4, K71.1, K72.1, K72.9, K76.5, K76.6, K76.7 | |
| Metastatic solid tumour | C77.x–C80.x | |
| AIDS/HIV | B20.x–B22.x, B24.x | |

ATC: Anatomical therapeutic chemical classification; AIDS: Acquired immunodeficiency syndrome; CDI: *Clostridioides difficile* infection; HIV: Human immunodeficiency virus; ICD-10: International classification of diseases; ICD-10-GM: International classification of diseases, German modification

**^a^** Adapted from Dihn et al. 2019 [1].

**^b^** Definition of comorbidities is based on Quan et al., 2005 [2].

**Table S2** Attrition table

| **Selection of patients** | **N (%)** |
| --- | --- |
| Patients with a record of a CDI episode in a hospital or community**^a^** setting | 12,330 (100.0) |
| Patients with complete key demographics | 12,330 (100.0) |
| Patients aged ≥18 years on index date | 12,101 (98.1) |
| 360 days of continuous enrolment in the database prior to index date | 11,939 (98.7) |
| Patients with no prior CDI during 60-day pre-index period | 11,884 (99.5) |
| **Total number of patients for cross-sectional analysis** | **11,884** |
| **Hospitalized CDI patients** | **10,491 (88.3%)** |
| **Community-treated CDI patients** | **1,393 (11.7%)** |

**^a^** Patients with an index CDI episode reported in community setting who presented with ≥1 gastrointestinal condition during the

same or the previous quarter of the CDI or who had no record of biological tests for identification of bacterial toxin A or B nor

prescriptions of antibiotics indicated for CDI were also excluded.

**Figures**

**
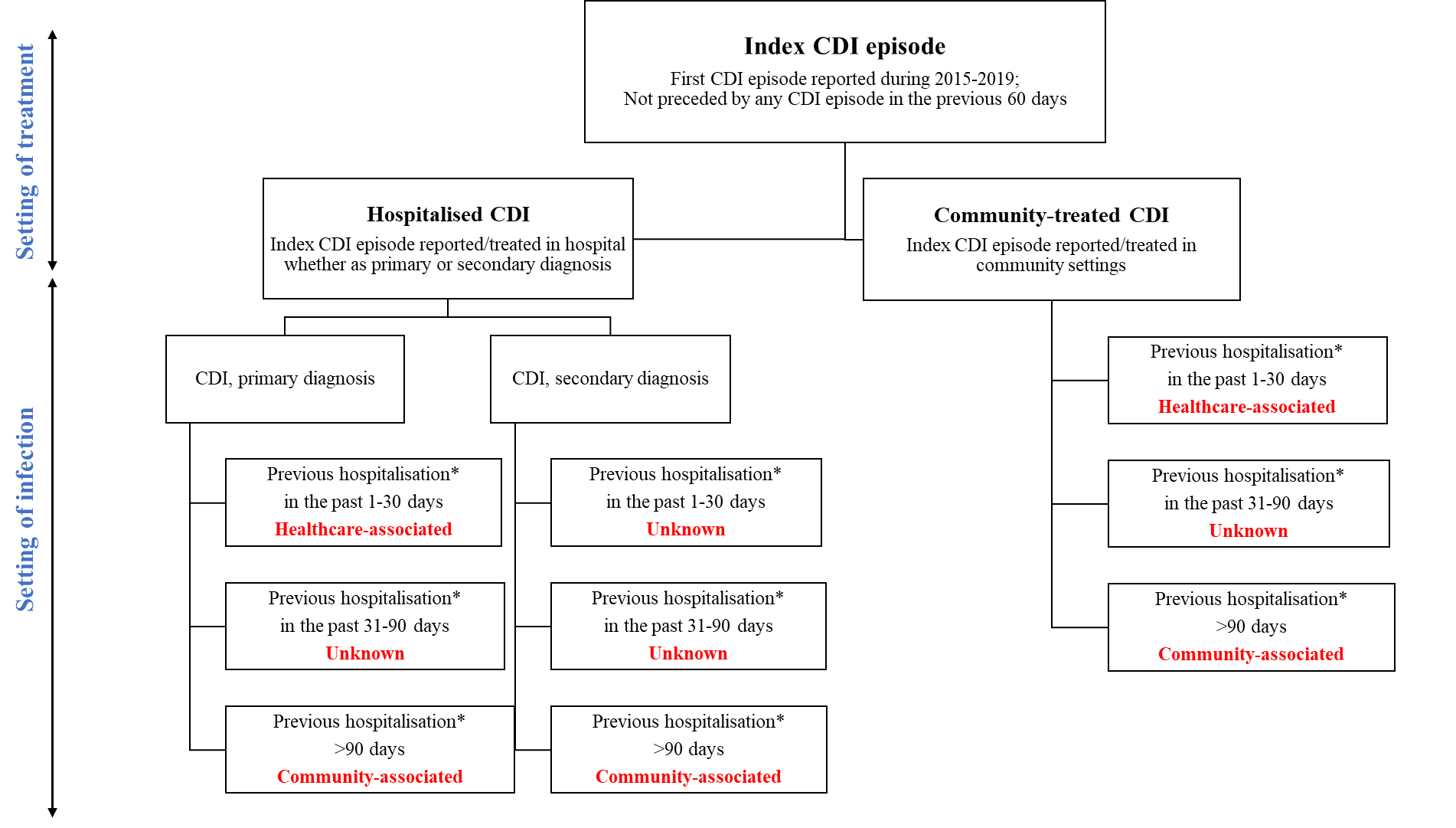
**

*Previous hospitalisation includes complete hospitalisation (>24 h or overnight stay, irrespective of cause) or transfer from a healthcare facility.

**Fig. S1** Classification of index CDI episodes by setting of treatment and infection.

CDI: *Clostridioides difficile* infection


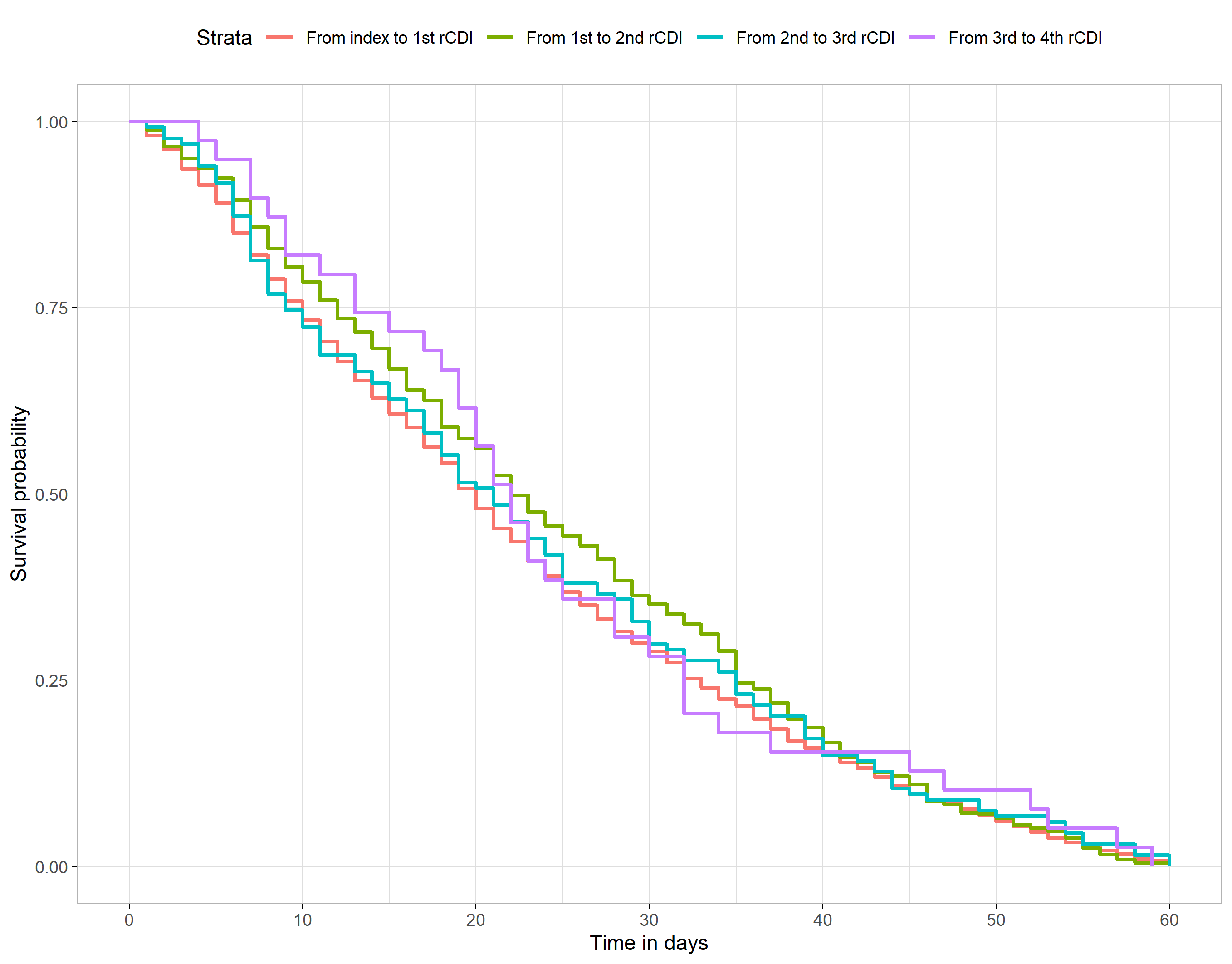


**Fig. S2**  Time from index CDI to first rCDI and from previous rCDI to next rCDI.

CDI: *Clostridioides difficile* infection; rCDI: Recurrent *Clostridioides difficile* infection

**References**

1. Dinh A, Le Monnier A, Emery C, Alami S, Torreton E, Duburcq A, Barbier F. Predictors and burden of hospital readmission with recurrent Clostridioides difficile infection: a French nation-wide inception cohort study. Eur J Clin Microbiol Infect Dis. 2019;38(7):1297-1305.

2. Quan H, Sundararajan V, Halfon P, Fong A, Burnand B, Luthi JC, et al. Coding algorithms for defining comorbidities in ICD-9-CM and ICD-10 administrative data. Med Care. 2005;43(11):1130-1139.
